# Development of the self-management scale for urolithiasis patients with indwelling double-J tube

**DOI:** 10.1007/s00345-024-04797-6

**Published:** 2024-03-06

**Authors:** Ziqi Hu, Aoli Huang, Ruiyao He, Fangxin Wei, Yu Wang

**Affiliations:** 1 School of Nursing, Jinan University, Guangzhou, 510630 Guangdong China; 2 Department of Cardiac Vascular Surgery, The First Affiliated Hospital of Zhejiang University, Hangzhou, 311100 Zhejiang China; 3 The Community Health Service Center of Jinan University, The 1st Affiliated Hospital of Jinan University, Nursing College of Jinan University, Guangzhou, 510630 Guangdong China

**Keywords:** Urolithiasis, Double-J tube, Self-management, Scale

## Abstract

**Purpose:**

To develop a specific self-management scale applicable to patients with indwelling double-J tube in urolithiasis, and to test its reliability and validity.

**Methods:**

The construction and validation of our scale involved three stages. First, an initial version of the questionnaire was formed through literature analysis, group discussions, semi-structured interviews, and the Delphi method. Second, a pre-survey was conducted with 20 urolithiasis patients with indwelling double-J stent placement to test their understanding of the initial questionnaire items and its acceptability. Finally, a formal survey of 234 patients with indwelling double-J tube for urolithiasis was conducted, and the scale was tested for reliability and validity.

**Results:**

After the three stages, a specific self-management scale for urolithiasis patients with indwelling double-J tube was developed, consisting of 30 items across five dimensions with a cumulative contribution rate of 52.541%. The content validity index for item level ranged from 0.8 to 1, and the content validity index for the questionnaire level was 0.93. The correlation between each item and its dimension was > 0.4. The Cronbach’s alpha coefficient for the overall questionnaire was 0.910, and the Cronbach’s alpha coefficients for each dimension ranged from 0.672 to 0.865. The split-half reliability of the overall questionnaire was 0.864, and the split-half reliabilities for each dimension ranged from 0.659 to 0.827. The test–retest reliability of the overall questionnaire was 0.840, and the test–retest reliabilities for each dimension ranged from 0.674 to 0.818.

**Conclusion:**

The specific self-management scale for urolithiasis patients with indwelling double-J tube has good reliability and validity, and it is a reliable and effective tool for evaluating and assessing the self-management level of patients with indwelling double-J tube in urolithiasis.

## Introduction

Nephrolithiasis, also known as urinary stone disease, is one of the most common diseases in urology. The incidence of urinary stone disease is between 2 and 20% in different regions of the world [1]. The incidence of urinary stone disease in adults in China is about 6.5% [2], and the recurrence rate is high, reaching up to 50% [3].Stone recurrence can lead to complications such as recurrent urinary tract infections, urinary tract obstruction, and even adverse consequences such as renal dysfunction and nephrectomy, bringing great pain to patients [4, 5].

Double-J stent is a commonly used auxiliary tool in the surgical treatment of urolithiasis, and its clinical application is becoming more and more widespread [6]. During the period of double-J stent placement, patients often experience bladder irritation, gross hematuria, back pain, displacement or dislodgement of the stent and other complications, with a high incidence rate of about 60–80%, which increases the patient ‘s hospitalization time and economic burden, and also causes varying degrees of negative emotions such as anxiety and fear, affecting the patient’s quality of life [7–10]. Patients with urolithiasis who have double-J stents have an important role in preventing or reducing stent-related complications and stone recurrence through good self-management [11, 12]. Understanding and comprehensively evaluating the self-management status of patients with ureteral stents due to urolithiasis, including symptom observation and coping, adherence to medical advice, dietary and fluid management, activity and excretion management, psychological and social management, and access to disease information, is of great significance for healthcare workers to develop targeted interventions and carry out targeted health education to help patients prevent complications of ureteral stents and stone recurrence.

However, there is currently a lack of a specific self-management assessment tool for patients with ureteral stents due to urolithiasis. The purpose of our study is to develop a new instrument to assess the self-management level of patients with ureteral stents due to urolithiasis.

## Materials and methods

Convenience sampling was used to select patients from the Department of Urology of a tertiary hospital in Guangzhou, China for a questionnaire survey from May 2021 to November 2021. Prior to participant recruitment and data collection, the study protocol was approved by the Local Ethics Committee (KY-2022–087).

*The inclusion criteria for the study are as follows*: (1) age ≥ 18 years; (2) patients diagnosed with urolithiasis. (3) presence of double-J stent; (4) voluntary participation in the study and signing of the informed consent form.

*The exclusion criteria are as follows*: (1) malignant tumors invading the urinary system; (2) cognitive impairment or mental illness which prevents normal communication; (3) physical weakness and inability to complete the questionnaire.

### Literature review, group discussion, semi-structured interview

We conducted a literature review on databases including CNKI, VIP, PubMed, Web of Science, Medline, CINAHL, and Embase. The literature review was conducted up to March 2021. All databases were searched using keywords such as “urinary calculi”, “urinary calculus”, “urinary stone”, “ureteral stent”, “double j tube”, “double j stent”, and “self-management”. In addition, patients meeting inclusion criteria were interviewed. The following is an outline of the interview:

- ① How do you observe and manage discomfort symptoms during the period of indwelling double-J stent?
- ② After discharge, in order to protect the double-J stent and avoid stone recurrence, how do you take care of yourself?
- ③ How was your mood during the period of indwelling double-J stent after discharge? How did you interact with your family and friends?
- ④ When you have problems related to urolithiasis or double-J stent, what methods do you take to solve them?

### Theoretical basis

The theoretical framework of this study was constructed based on the social cognitive theory [13], self-efficacy theory [14], and self-determination theory [15].

### Theoretical framework

Based on the social cognitive theory of human subjectivity and individual self-management model, by providing relevant health education guidance to patients, we can make them actively acquire disease-related information and pay attention to the self-observation of disease-related symptoms and self-response to the emergence of symptoms. Based on the idea that self-beliefs in self-efficacy theory can regulate individual behaviors to improve self-management, we can help patients establish good self-efficacy, which can be transformed into confidence in treating the disease, preventing complications, and regulating bad emotions. Based on the three basic psychological needs of competence, relationship, and autonomy in the self-determination theory, we can help urolithiasis patients with indwelling double-J tubes to respond to the needs of the disease treatment, cooperate with the medical and nursing care during the treatment of the disease, cultivate good habits in daily activities, diet and water intake, and guide the patients to regulate their family and social relationships to cope with their bad moods.

### Using the Delphi method to construct an initial scale

33 clinical and nursing experts in the field of urology were invited to participate in 2 rounds of expert consultations. Through discussions and modifications of the items in the scale, we aimed to construct a self-management initial scale for patients with ureteral stent placement due to urinary stones.

### Feasibility test

To evaluate the practical application of the questionnaire, a preliminary survey would be conducted using the initial questionnaire on 20 patients with ureteral stent placement who were followed up at the urology outpatient clinic.

### Formal investigation

We conducted a formal investigation in the Department of Urology of the hospital from June 2021 to November 2021 to develop the final version of the scale and test its reliability and validity.

### Statistical analysis

Statistical analysis was performed using SPSS 27.0 software. Count data were described using frequency and percentage, while measurement data were described using mean ± standard deviation (± S). Item analysis was carried out using item analysis, coefficient of variation, critical ratio (independent sample t-test), Pearson correlation analysis, and Cronbach’s α coefficient method. Exploratory factor analysis was performed using principal component analysis to test construct validity. Expert evaluation was used to test content validity, with the content validity index (I-CVI) at the item level and the scale level (S-CVI) as the evaluation indicators. Reliability analysis was conducted using Cronbach’s α coefficient, split-half reliability, and test–retest reliability.

## Result

### Formation of the initial scale

After two rounds of Delphi expert inquiries, one item was deleted, two items were merged and six items were added. The revised questionnaire consisted of five dimensions: disease treatment management, diet and water management, activity and excretion management, psychosocial management, and disease information management, with 9, 10, 9, 4, and 4 items, respectively, totaling 36 items.

### Official investigation results

#### General information

A total of 234 eligible patients were collected, as shown in Table 1 for specific details.

**Table 1:** General information of the subjects of the scale study

Parameters	Overall (*n* = 234)
Gender, *n* (%)	
Male	140 (59.8)
Female	94 (40.2)
Age	
20 ~ 40	68 (29.1)
41 ~ 60	106 (45.3)
61 ~	60 (25.6)
Marital status	
Unmarried	32 (13.7)
Married	193 (82.5)
Divorce	9 (3.8)
Family living situation	
Living with partner or children	200 (85.5)
Living alone	34 (14.5)
Education level	
Junior high school and below	86 (36.8)
High school/junior high school	59 (25.2)
College	47 (20.1)
Bachelor’s degree or above	42 (17.9)
Career	
Farmers	38 (16.2)
Students	3 (1.3)
Public officials	57 (24.4)
Individuals	23 (9.8)
Retirement	62 (26.5)
Other	51 (21.8)
Labor intensity	
Mental work	57 (24.4)
Light manual labor	160 (68.4)
Heavy physical labor	17 (7.3)
Monthly income (yuan)	
≤ 3000	64 (27.4)
3001 ~ 6000	75 (32.1)
6001 ~ 12,001	70 (29.9)
> 12,001	25 (10.7)
Residence	
City	131 (56.0)
Rural	103 (44.0)
Medical insurance	223 (95.3)
Length of time with urolithiasis	
≤ half year	38 (16.2)
≤ 2 years	42 (17.9)
≤ 3 years	35 (15.0)
> 3 years	119 (50.9)
Number of reviews	
0 times	6 (2.6)
1 time	24 (10.3)
2 time	98 (41.9)
3 time	106 (45.3)
Length of retention of double-J tube	
≤ half a month	12 (5.1)
≤ 1 month	163 (69.7)
≤ 2 months	47 (20.1)
> 3 months	12 (5.1)
Complication	
Diabetes, *n* (%)	27 (11.5)
High blood pressure, *n* (%)	66 (28.2)

Other means all occupations other than those listed in the table

#### Selection of scale items

The item analysis results showed that the option selection rates for all 36 items were less than 80%, indicating good concentration of the item options. The results of coefficient of variation method showed that the coefficient of variation of two items was less than 0.15, indicating poor discrimination, so deleted. Independent sample *t*-tests were used, and the results showed that the P values were less than 0.05, and the critical values (t values) were greater than 3.00, indicating good discriminant validity.

Pearson correlation analysis showed that the correlation coefficients of all items were greater than 0.3 and P values were less than 0.05. The Cronbach’s α coefficient method showed that the Cronbach’s α coefficient of the scale was 0.914, and the Cronbach’s α coefficients of each dimension ranged from 0.685 to 0.850. After deleting three items, the Cronbach’s α coefficients of the total scale and each dimension increased, so they were deleted.

### Validity analysis results

#### Content validity

In this study, content validity was calculated based on the importance ratings of the scale by experts in the second round of consultations. The general evaluation criteria were I-CVI > 0.78 and S-CVI > 0.90 for good content validity. The results showed that the item-level I-CVI ranged from 0.8 to 1 and the scale-level S-CVI was 0.93.

#### Construct validity

**Fig. 1:**
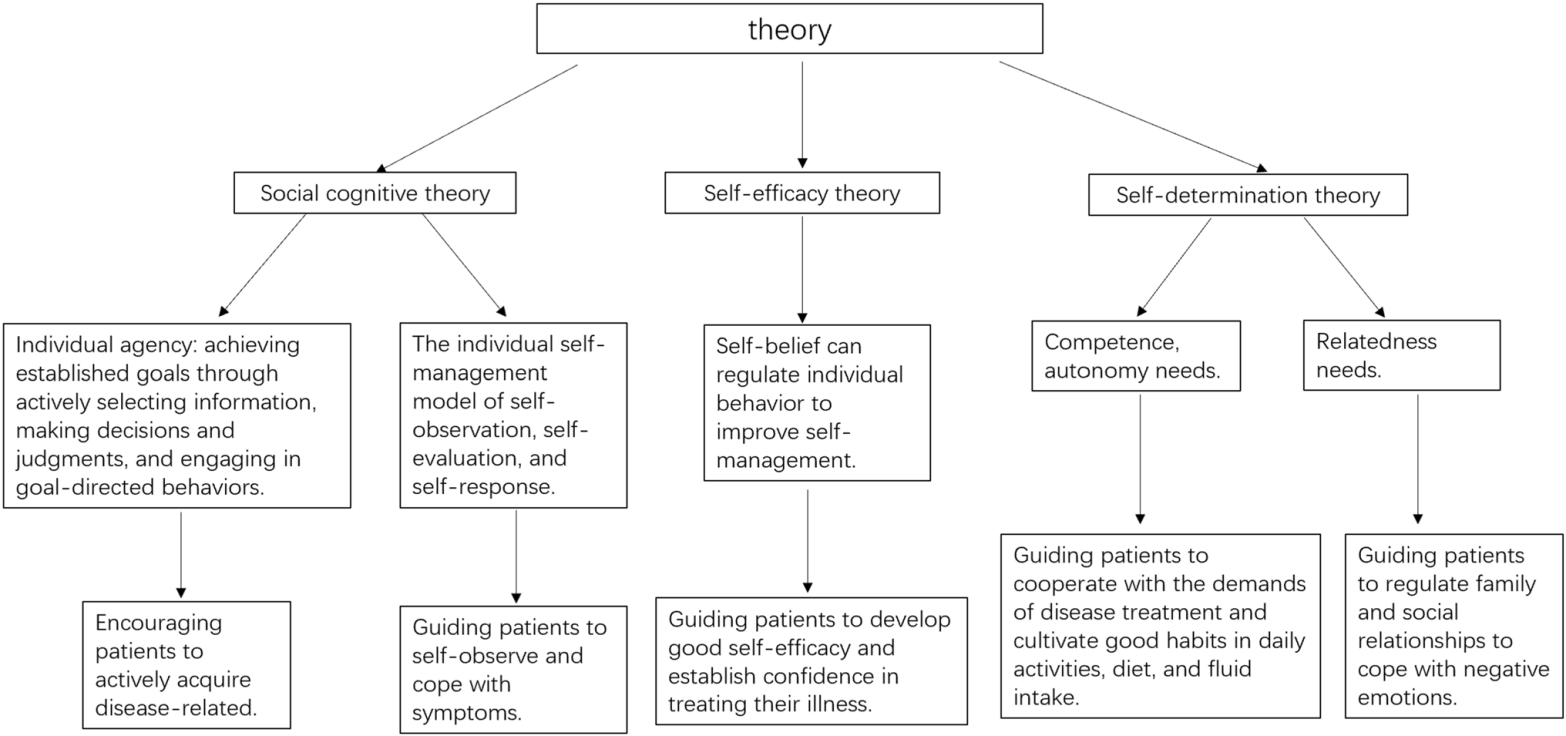
Results of the third exploratory factor score of the gravel plot test

Exploratory factor analysis (EFA) was used to test the construct validity. The calculated KMO value was 0.896, and the Bartlett sphere test χ2 value was 2878.930 with *P* = 0.000, indicating that factor analysis was appropriate. After limiting the extraction to five common factors, one item was found to have a cross-loading, with factor loadings greater than 0.400 on two or more common factors, and was removed. Exploratory factor analysis was conducted again, and the results showed a KMO value of 0.893, and a Bartlett sphere test χ2 value of 2742.186, with all item communalities greater than 0.2. The scree plot test results showed that the slope line became flat from the fifth factor onwards, indicating that no special factors were worth extracting. The total variance explained by the five common factors was 52.541%, all factor loadings of all items were greater than 0.400. Please refer to Fig. 1, Table 2, 3 and 4 for details.

**Table 2:** Results of the commonality test for the third factor analysis

Entry	Initial	Extraction	Entry	Initial	Extraction
A1	1.000	0.607	B9	1.000	0.597
A2	1.000	0.604	B10	1.000	0.427
A3	1.000	0.736	C1	1.000	0.516
A4	1.000	0.776	C2	1.000	0.473
A5	1.000	0.585	C5	1.000	0.445
A6	1.000	0.434	C6	1.000	0.694
A8	1.000	0.454	C7	1.000	0.535
A9	1.000	0.431	C8	1.000	0.563
B1	1.000	0.330	D1	1.000	0.443
B2	1.000	0.524	D2	1.000	0.582
B3	1.000	0.697	D3	1.000	0.507
B4	1.000	0.472	D4	1.000	0.562
B5	1.000	0.357	E1	1.000	0.517
B6	1.000	0.488	E3	1.000	0.566
B7	1.000	0.383	E4	1.000	0.459

**Table 3:** The third factor analysis extracts the factors with eigenvalues greater than 1

Ingredients	Initial eigenvalue			Sum of squares of extracted loadings			Sum of squared rotating load volumes		
	Total	Variance contribution rate	Cumulative variance contribution rate	Total	Variance contribution rate	Cumulative variance contribution rate	Total	Variance contribution rate	Cumulative variance contribution rate
1	8.601	28.670	28.670	8.601	28.670	28.670	4.281	14.270	14.270
2	3.024	10.081	38.751	3.024	10.081	38.751	4.261	14.202	28.472
3	1.558	5.193	43.944	1.558	5.193	43.944	2.572	8.574	37.046
4	1.516	5.055	48.999	1.516	5.055	48.999	2.499	8.330	45.375
5	1.063	3.542	52.541	1.063	3.542	52.541	2.150	7.165	52.541

**Table 4:** Factor loading matrix after the third factor analysis rotation

Entry	Ingredients				
	1	2	3	4	5
A1	0.718				
A2	0.706				
A3	0.846				
A4	0.806				
A5	0.672				
A6	0.428				
A8	0.404				
A9	0.513				
B1		0.417			
B2		0.650			
B3		0.806			
B4		0.636			
B5		0.521			
B6		0.605			
B7		0.543			
B9		0.751			
B10		0.487			
D1			0.534		
D2			0.616		
D3			0.613		
D4			0.669		
C1				0.461	
C2				0.482	
C5				0.447	
C6				0.691	
C7				0.679	
C8				0.640	
E1					0.539
E3					0.605
E4					0.521

#### Reliability analysis results

The results of this study showed that the Cronbach’s α coefficient of the total scale was 0.910, and the Cronbach’s α coefficients of each dimension ranged from 0.672 to 0.865. The split-half reliability of the total scale was 0.864, and the split-half reliabilities of each dimension ranged from 0.659 to 0.827. The test–retest reliability of the total scale was 0.840, and the test–retest reliabilities of each dimension ranged from 0.674 to 0.818. See Table 5 for details.

**Table 5:** Total table and Cronbach’s alpha coefficient for each dimension

Dimensionality	Number of entries	Cronbach’s α
Total table	30	0.910
Disease treatment management	8	0.865
Diet and water management	9	0.823
Psychosocial management	4	0.723
Active excretion management	6	0.734
Disease information management	3	0.672

## Discussion

We developed a self-management scale for urolithiasis patients with indwelling double-J tube based on self-management theory. The scale was created through literature review, group discussion, semi-structured interviews, and the Delphi method. We tested the reliability and validity of the scale, which covers five dimensions: disease treatment, diet and water intake, psychosocial factors, activity and excretion, and disease information, with a total of 30 items.

Currently, self-management-related scales for urolithiasis patients included the Self-Management Behavior Scale for Patients with Recurrent Urinary Stones [16], the Self-Management Competency Evaluation Scale for Patients with Urolithiasis [17], the Self-Management Questionnaire for Patients with Upper Urinary Tract Stones [18], the Self-Management Competency Survey Scale for Patients with Urolithiasis [19], the Compliance Survey Questionnaire for Patients with Recurrent Renal Stones [20], the Self-administered Questionnaire for Patients with Renal Stones [21], the Wisconsin Stone Quality of Life questionnaire [22], the Cambridge Ureteral Stone Patient Reported Outcome Measurement Tool [23] and the Cambridge Renal Stone Patient Reported Outcome Measurement Tool [24].All such scales were applied to patients with urolithiasis, not patients with indwelling double-J tubes for urolithiasis. The two differ in self-management of daily activities and psychosocial aspects. In terms of daily activities, ordinary urolithiasis patients, if the treatment of the disease allows, can promote stone removal by jumping and increasing the amount of activity, while urolithiasis patients with indwelling double-J tubes will be induced to hematuria, stent tube displacement or dislodgement and other complications if they do not move properly during the period of insertion of the tubes (sudden squatting, bending, abdominal or lumbar and other forceful movements, running, rapid up and down the stairs and other strenuous exercises); in terms of the psychosocial aspect, the insertion of the double-J tubes can aggravate the urolithiasis, which can be caused by the complications. In the psychosocial aspect, indwelling double-J tube can aggravate the psychological burden of urolithiasis patients, causing negative emotions such as anxiety and fear, which affects the quality of life [8], so it is more necessary for urolithiasis patients with indwelling double-J tubes to improve their confidence in coping with the disease, and to carry out good self-management through the help of their family members and friends. Therefore, such scales used to assess self-management in patients with indwelling double-J tubes for urolithiasis lack specificity. Therefore, this study developed a specialized assessment tool to assess self-management in these patients.

In addition, with the current self-management scales for ureteral stone patients with double-J stent placement, such as the Ureteral Stent Related Symptom Questionnaire (USSQ) [25]. It mainly contained dimensions such as urinary symptoms, body pain, general health, work performance, sex life, and additional questions. This questionnaire was self-assessed by patients and is scored on a Likert scale of 5 and 7. Cronbach’s alpha coefficient was 0.7 and retest reliability is 0.84, which was good reliability and validity. This scale has been able to assess the management of disease symptoms in patients with indwelling ureteral stents, but it lacked the assessment of diet and water, psychosocial, and disease information in patients with urolithiasis with indwelling double-J tubes. Therefore, the scale developed in this study contains more comprehensive and enriched dimensions.

In summary, this scale has a certain rigor and scientificity, and can effectively reflect the level of self-management of urolithiasis patients with indwelling double-J tube. At the same time, this scale has high practical value in both clinical and research fields. First, by accurately assessing the self-management level of patients with ureteral calculi and JJ stent placement, it can effectively predict and explain the relevant factors that affect their self-management level, and help medical staff provide targeted health education for such patients. Second, it can help patients prevent complications of JJ stent placement and stone recurrence, and provide new ideas for developing interventions to improve self-management levels.

### Strengths and limitations

Due to time and other constraints, this study only conducted a questionnaire survey in a tertiary hospital in Guangzhou, and the representativeness of the sample was limited. It is recommended that future research should conduct large-scale questionnaire surveys in multiple centers in various regions across the country to increase the representativeness of the sample. In addition, validation factor analysis was not conducted in this study, and in the future, sufficient samples should be collected to conduct validation factor analysis to further validate and improve the scale.

## Conclusion

The specific self-management scale for urolithiasis patients with indwelling double-J tube has good reliability and validity, and it is a reliable and effective tool for evaluating and assessing the self-management level of patients with indwelling double-J tube in urolithiasis.

## Data Availability

To protect patient privacy and security, the data in this article cannot be shared.

## Appendix (see Table 6)

**Table 6:** The following entries describe your self-management during the treatment of stones with indwelling double J-tubes. Please read the content of each entry carefully, choose the one you think is the most appropriate out of the 5 options according to your actual situation, and tick the corresponding number. There is no right or wrong answer, you can only choose one option for each entry, please don't make more than one choice or omit to choose. hasn't represents 1 mark, less represents 2 marks, now and then represents 3 marks, regular represents 4 marks, always represents 5 marks. The total score is 30–150 points, the higher the score, the higher the level of self-management

Item	Hasn’t	Less	Now and then	Regular	Always
Disease treatment management	Options				
I will self-observe whether there are symptoms such as frequent urination, urgent urination, painful urination, hematuria, dripping sensation after urination, and pain in the lower back and abdomen					
If these symptoms occur, I will relieve them by bed rest, reducing activity, and increasing water intake					
If the above symptoms are not relieved by measures and persist, I will contact my primary care physician or return to the hospital for consultation					
If I find double-J prolapsing out of the urethra, I will seek prompt medical attention					
I will contact my primary care physician or return to the hospital if there is a persistent fever of unknown origin					
I will follow the doctor’s instructions to take regular medication at regular intervals					
I focus on preventing disease and strengthening my body resistance					
I will return to the hospital for treatment and extubation within the time specified by the attending physician					
Diet and water management	Options				
If the condition allows, I can drink up to 2500 ~ 3000 ml of water per day (about 5 ~ 6 bottles of 500 ml mineral water)					
I will eat less high-purine food, such as old fire soup (soups made with high-purine meats such as chicken, duck and lamb), animal offal, and all kinds of seafood					
I will eat less high-sodium food, such as pickles (pickles fermented by pickling with salt and seasonings) and soy sauce (sauce made from boiled soybeans), and control the daily salt intake to 6 g (about the amount spread over a beer cap)					
If you are overweight or obese, I would eat less high-fat food, such as chocolate, fatty meat, fried food, and Western fast food					
I will eat less food high in oxalic acid, such as spinach, celery, tomatoes, grapes, strawberries, mangoes, and all kinds of nuts					
I do not intentionally increase the intake of foods rich in animal protein (animal meat, eggs, etc.) when I eat them normally					
I will drink less strong tea, coffee, alcohol, vitamin C drinks, carbonated drinks (Coke, Sprite)					
I will avoid excessive intake of spicy, irritating foods, such as chili peppers, pepper, ginger and garlic					
I will eat more high-fiber foods, such as fresh vegetables and fruits					
Psychosocial management	Options				
I will urinate in time when there is your desire to urinate, not to hold urine					
I will pay attention to the prevention of constipation, keep the bowels open					
I will pay attention to the appropriate change of position, avoid prolonged sitting, prolonged standing, prolonged squatting					
I will avoid abdominal exertion, such as bending over to lift heavy objects, holding children, sudden squatting, violent sneezing, coughing					
I will avoid doing large limb and waist stretching movements and exercises, such as stretching, swimming					
I will avoid strenuous exercise, such as running, fast walking up and down stairs, playing ball, riding a bicycle for a long time					
Active excretion management	Options				
I believe that good self-management can prevent the complications of indwelling double-J tube and reduce the recurrence of stones					
I have confidence in good self-management					
I will self-regulate if I am afraid or anxious due to complications of indwelling double-J tube or restriction of activities					
If I cannot relieve my fear or anxiety through self-regulation, I will seek help from others					
Disease information management	Options				
I will keep information about the disease during treatment					
If any questions arise, I will consult with the medical staff					
I will learn about urolithiasis and double-J tube through internet, TV, lectures, etc					
